# Predictive factors of treatment success in two-dose methotrexate regimen in ectopic tubal pregnancy: A retrospective study

**DOI:** 10.12669/pjms.37.5.4299

**Published:** 2021

**Authors:** Caglar Helvacioglu, Keziban Dogan

**Affiliations:** 1 Dr. Caglar Helvacioglu Department of Obstetrics and Gynecology, Health Sciences University, Umraniye Training & Research Hospital, Istanbul, Turkey; 2 Dr. Keziban Dogan Department of Obstetrics and Gynecology, Health Sciences University, Bakirkoy, Dr. Sadi Konuk Training and Research Hospital, Istanbul, Turkey

**Keywords:** pregnancy, ectopic, methotrexate, therapeutics

## Abstract

**Objectives:**

To investigate the predictive factors of success or failure in treating ectopic tubal pregnancies with two-dose methotrexate (MTX).

**Methods:**

The records of patients treated for tubal EP with two-dose MTX were retrospectively reviewed. Patients were divided into two groups; the Group-I (failure) consisted of patients who did not respond to MTX therapy and the Group-II (success) included patients who were successfully treated with MTX. Parameters, including the week of gestation, presence or absence of fetal cardiac activity, gestational sac size, serum β-hCG levels, and adverse effects were compared.

**Results:**

Fifty patients were included in this study, 8 (16%) were in Group-I and 42 (84%) were in Group-II. Patients in Group-I required surgery after a mean duration of 6.7±3 days after administering the initial dose of MTX. There was no difference between the groups in terms of the week of gestation, presence or absence of fetal cardiac activity, gestational sac size, serum β-hCG levels, and adverse effects. The average time to β-hCG negativization was 31 days in Group-II.

**Conclusions:**

The two-dose MTX protocol has a reasonable success rate, which seems to be dependent on serum β-hCG levels.

## INTRODUCTION

Ectopic pregnancy is encountered in 2.6% of all pregnancies and occurs when the fertilized ovum is implanted outside of the endometrial cavity_._^1^ The fallopian tube is the most common location for an EP. It is widely accepted that EP is one of the major causes of maternal morbidity and mortality in the first trimester of pregnancy, despite improved diagnostic methods leading to early diagnosis and treatment.^2^,^3^ Transvaginal ultrasonography helps in the early diagnosis of EP, and intramuscular methotrexate (MTX) is frequently used for the medical management of unruptured EP.^4^,^5^ Methotrexate works by inactivating dihydrofolate reductase, which is required for DNA and RNA synthesis.^6^ Although its mechanism of action is known in detail, there is no consensus regarding the treatment protocol and surveillance guidelines. There are currently different MTX treatment protocols (single-dose, hybrid, two-dose, and multiple-dose) in use to treat patients with EP.^7^ Several studies, including meta-analyses, worked on the determinants of MTX treatment success or failure in patients with EP.^7^-^10^ However, there in an insufficient number of studies to determine the effect of these parameters on the success rates of the two-dose MTX treatment protocol. In this study, we aimed to determine the predictive factors of success in treating unruptured EP with a two-dose MTX regimen.

## METHODS

The ethical review committee of University of Health Sciences Bakirkoy Dr. Sadi Konuk Training and Research Hospital approved the study (2016/02/11). All participants gave verbal and written consent before enrollment in the study. The medical records of the women admitted to our obstetric tertiary care center with a diagnosis of EP between January 2013 and October 2015 were retrospectively reviewed. Patients diagnosed as having unruptured tubal EP, hemodynamically stable during admission, and who received two-dose MTX treatment were included. Patients who were given single-dose or multiple-dose MTX treatment regimens, hemodynamically unstable patients, patients who underwent surgery for EP before receiving medical treatment, and patients with contraindications to MTX therapy were excluded. Also, patients who did not adhere to the treatment or follow-up protocol were not included in the study.

Demographic data included age, body mass index (BMI), gravidity, smoking history, marital duration, history of abortion, prior EP, history of intrauterine device use, primary symptom on presentation (abdominal pain or vaginal bleeding), size of EP (mm) presence or absence of fetal cardiac activity, endometrial thickness (mm), duration of hospital stay, day of rupture, and serum β-human chorionic gonadotropin (hCG) levels on days 0, four, and seven were recorded. All study participants received intramuscular MTX at a dose of 50 mg/m^2^ body surface area that was calculated based on height and body weight using a nomogram. A repeat dose was given four days after the initial dose. Negativization of β-hCG without undergoing surgery was defined as treatment success. Surgical treatment was performed in patients who did not respond to MTX treatment or had a tubal rupture after medical treatment. Tubal rupture was diagnosed based on hemodynamic, clinical, and radiologic signs such as rapid blood pressure drop, increased abdominal pain, and blood detection in the abdominal cavity, as confirmed using ultrasound.

Patients were divided into two groups; the failure group and the success group. The failure group consisted of patients who could not be successfully treated with MTX. These patients were initially treated with MTX but consequently underwent surgery either due to unresponsiveness to medical treatment or tubal rupture. The success group comprised patients who were successfully treated with two-dose MTX therapy. Negativization of serum β-HCG levels without surgery was defined as treatment success. The adverse effects of MTX, as well as liver and kidney function, were checked in each participant one week after completing treatment and patients were asked for complications such as stomatitis, conjunctivitis, nausea, vomiting, and gastrointestinal symptoms.

Statistical analyses were performed using the SPSS 15.0 software (SPSS Inc., Chicago, IL). Descriptive data are given as means and percentage distributions. The normality of data distribution was tested using the Kolmogorov-Smirnov test. The Mann-Whitney U test was used to determine the differences between the means of the groups, and Pearson’s Chi-square test and Fisher’s exact test were used to compare the differences between percentages. P-values were considered statistically significant when they were lower than 0.05.

## RESULTS

The mean patient age of the cohort was 31.7 (range, 20-52) years. The mean BMI and marital duration were 25.6 (range, 17.5 - 39) kg/m^2^ and 7.3 (range, 0-25) years. Eighteen of the 50 (36%) study patients were smokers. The median gravidity and parity numbers of the patients were 3 (range, 1-7) and 1 (range, 0-5), respectively. Thirteen of 50 (26%) study participants had a history of prior EP, endometriosis, infertility or pelvic infection.

Our analysis revealed that 42 of the 50 (84%) study patients were successfully treated, and eight (16%) patients developed tubal rupture or needed surgical treatment. There were eight patients in the failure group (Group-I) and 42 patients in the success group (Group-II) (Table-I). A comparison of the parameters between the two groups is presented in Table-I. There was no difference between the groups in terms of age, BMI, smoking history, gravidity and parity numbers, history of intrauterine device use, abdominal pain, vaginal bleeding, endometrial thickness (mm), EP size (mm), fetal cardiac activity, free fluid in the Douglas pouch, tubal rupture duration (days), length of hospital stay (days), and adverse effects including nausea and abdominal pain not requiring medication.

**Table-I:** Comparison of the parameters between the two groups.

	Failure Group	Success Group	*P-value*

	n=8	n=42	

	16%	84%	
Age	29.5 (20-37)	31 (20-52)	0.458
BMI	25.3 (17.5-39)	24.1 (20-28.3)	0.490
Gravidity	3 (1-7)	2.5 (1-5)	0.969
Smoking history	3 (37.5%)	15 (35.7%)	0.999
Duration of marriage (years)	1.5 (0.9)	6.5 (0-25)	0.031*
Abortion or curettage	5 (62.5%)	13 (31%)	0.118
EP history	2 (25%)	6 (14.3%)	0.485
IUD	1 (12.5%)	14 (33.3%)	0.407
Pain	6 (75%)	29 (69%)	0.999
Vaginal bleeding	6 (75%)	28 (66.7%)	0.999
Endometrial thickness (mm)	7 (6-10)	8 (3-18)	0.490
Size of EP (mm)	21.25 (14-30.5)	16.25 (0-32.5)	0.137
Fetal cardiac activity	1 (12.5%)	3 (7.1%)	0.514
Free Fluid in the Douglas pouch	5 (62.5%)	13 (31%)	0.118
Duration of tubal rupture (days)	6.7±3	-	-
Duration of hospitalization (days)	3.9±1.6	4.6±2.2	0.474
Adverse effects	1 (12.5%)	5 (11.9%)	0.999

	n=8	n=42	

β-hCG level at D0 (IU/L)	4465 (1384-15123)	2825 (244-12153)	0.015*
	n=7	n=42	

β-hCG level at D4 (IU/L)	4806 (2094-17031)	2930 (166-10723)	0.012*

	n=3	n=42	

β-hCG level at D7 (IU/L)	6271 (2381-9867)	2422 (87-6823)	0.033*
Negativization of β-hCG (days)	-	31 (14-51)	

Significance level of

* p < 0.05

Mann-Whitney U test, Pearson’s Chi-square test and Fisher’s exact test,

BMI: Body mass index, EP: Ectopic pregnancy, IUD: Intrauterine device.

The median marriage duration in the failure group was significantly shorter than in the success group (p=0.031). Five of the 8 (62.5%) patients in the failure group had a history of abortion and 13 of the 42 (31%) patients in the success group had a history of abortion; however, the difference was not statistically significant (p>0.05).

Because patients who had EP rupture before day four or day seven were excluded from the comparative analysis of serum β-HCG levels, eight patients from the failure group were included in day 0, seven patients in day 4, and three patients were included in day 7 serum β-hCG level comparisons. By contrast, all 42 patients of the success group were included in the analysis. These comparisons revealed that all median serum β-hCG levels (i.e. day 0, day 4, and day 7) were significantly higher in the failure group than in the success group (p=0.015, p=0.012, and p=0.033, respectively). The mean time interval between day 0 and day of rupture was calculated as 6.7±3.

## DISCUSSION

This study was designed to determine the effectiveness of two-dose MTX and clinical factors influencing success in patients with EP. The therapeutic success of the two-dose protocol in our study was 84% and we detected mild adverse effects including nausea and abdominal pain that did not require medication. The therapeutic success rate of our study was higher than in the study by Saadati et al. (79%) and lower than that of Hamed et al. (88%).^11^,^12^ On the other hand, these researchers compared the success of single-dose and two-dose MTX and found that the success of two-dose MTX was higher, and reported no significant difference between the groups in terms of adverse effects.

Gupta et al. compared patients treated for EP with single-dose, two-dose or multidose MTX treatment protocols in a meta-analysis published in 2019, and they reported that the two-dose protocol was superior to the single-dose protocol in terms of treatment success, and its adverse effects were mild and temporary, similar to our results.^13^ Their findings also showed that patients with EP with relatively high serum β-hCG levels (3000 IU/L) and large adnexal masses (2 cm) had a better chance of responding to the two-dose MTX treatment protocol than the single-dose protocol. In line with this report, we found that the serum β-hCG levels in the failure group were higher compared with the success group. However, we found no association between treatment success and gestational sac size and the presence of fetal cardiac activity. However, some researchers had demonstrated that in 15-20% of cases where a single-dose MTX was used required a second dose of MTX if the decrease in β-hCG between days 4 and 7 was less than 15%, especially in patients with higher β-hCG levels.^14^,^15^

Our study limitations are being a retrospective study and the small sample size. Despite these limitations, the two-dose MTX protocol may be recommended as the first-line treatment, especially in patients with baseline β-hCG levels of >3000 IU/L or adnexal masses >2 cm, without an increase in adverse effects and no rescue dose of leucovorin used in the multiple-dose protocol. It is known that the rate of treatment failure and possibility of administering a second dose of MTX increase if the β-hCG level is above 2000 IU/L.^14^,^15^ In our study, patients with an average β-hCG level between 2825-4464 IU/L were successfully treated with the two-dose MTX protocol. We think that these results support the data of Gupta et al.^13^ In addition, the β-hCG negativization time can be extended from 35 days to 109 days in cases where a single-dose MTX protocol is used. We found that β-hCG negativization was 31 days in our success group. We think that shorter follow-up periods will enable patients to return to their routine more easily.^15^,^16^

Nevertheless, it should be kept in mind that patients with high serum β-hCG levels may have a greater possibility of requiring surgical intervention. In our study, the mean interval between the day of the first dose of MTX treatment and tubal rupture was 6.7±3 days. This finding implies that physicians following these patients should be aware of the risk of rupture, particularly during the first week after medical treatment.

## CONCLUSION

In conclusion, the intramuscular administration of two doses of MTX on days 0 and 4 is safe and effective for treating EP in selected patients. However, larger sample sizes are needed to support the study data.

### Authors Contribution:

**CH:** Conceived, designed, did statistical analysis & editing of manuscript, is responsible for integrity of the study. **KD:** Did data collection and manuscript writing. **CH & KD:** Did review and final approval of manuscript.
